# Prevalence and factors associated with intellectual impairment in children with epilepsy in two national referral hospitals in Uganda

**DOI:** 10.1186/s42494-026-00265-3

**Published:** 2026-07-01

**Authors:** Anita Arinda, Molly McVoy, Joan Abaatyo, Mark Kaddumukasa, Martha Sajatovic, Elly Katabira

**Affiliations:** 1 Department of Psychiatry, School of Medicine, College of Health Sciences, Makerere University, Kampala, 10022 Uganda; 2 Department of Psychiatry, University Hospitals of Cleveland, Case Western Reserve University School of Medicine, 11100 Euclid Avenue, Cleveland, OH USA; 3 Department of Psychiatry, Mulago National Referral Hospital, Kampala, 10023 Uganda; 4 Department of Psychiatry, King Ceasor University, Kampala, 10034 Uganda; 5 Department of Medicine, School of Medicine, College of Health Sciences, Makerere University, Kampala, 10022 Uganda; 6 Neurological and Behavioral Outcomes Center, University Hospitals Cleveland Medical Center & Case Western Reserve University School of Medicine, 11100 Euclid Avenue, Cleveland, OH USA

**Keywords:** Epilepsy, Children, Adolescents, Intellectual impairment, Uganda

## Abstract

**Background:**

Epilepsy is a common neurological condition in children and is often associated with intellectual impairment. However, there is limited information on the intellectual impairment among children with epilepsy in Uganda. This study assessed the prevalence and associated factors of intellectual impairment among children and adolescents with epilepsy attending two national referral hospitals in Uganda.

**Methods:**

This cross-sectional study included children and adolescents aged 5 to 17 years who had a diagnosis of epilepsy. Intellectual functioning was assessed using the Raven’s Progressive Matrices. Logistic regression analysis was performed to determine demographic and clinical factors associated with intellectual impairment.

**Results:**

Of 386 participants, 34.7% had intellectual impairment. Older age (adjusted odds ratio [aOR] = 6.94, *P* = 0.001), being in a special needs school (aOR = 14.50, *P* = 0.016), not being in school (aOR = 26.01, *P* < 0.001), delayed speech (aOR = 3.26, *P* = 0.030), and anti-seizure medication polypharmacy (aOR = 3.08, *P* = 0.020) were positively associated with intellectual impairment, while adolescent-onset epilepsy (aOR = 0.05, *P* = 0.030) was negatively associated.

**Conclusions:**

Intellectual impairment is common among Ugandan children and adolescents with epilepsy and is associated with older age, school non-attendance, special-needs school placement, delayed speech, and anti-seizure medication polypharmacy. These findings underscore the need for routine assessment of intellectual functioning and targeted interventions, including academic support, speech therapy, and optimization of treatment regimens.

**Supplementary Information:**

The online version contains supplementary material available at 10.1186/s42494-026-00265-3.

## Background

Epilepsy is a common neurological disorder in children and adolescents, with the highest burden in those living in low- and middle-income countries (LMICs) [1]. It is frequently linked with cognitive difficulties, including intellectual impairment [2]. Intellectual impairment refers to deficits in general mental capacity, such as reasoning, problem solving, planning, abstract thinking, judgment, and academic learning, confirmed by clinical assessment and standardized intelligence testing [3]. An interaction of multiple factors influences the relationship between epilepsy and intellectual impairment. The physiological factors that make the brain susceptible to seizures, for example, genetic and prenatal causes and perinatal risk factors, may also contribute to the development of intellectual impairment [4–6]. Prolonged and recurrent seizures in epilepsy can potentially harm brain development, structure, and function, resulting in intellectual impairment [7, 8]. Certain medications prescribed for seizure management have been found to induce substantial intellectual impairments [9, 10].

The co-occurrence of intellectual impairment with epilepsy has been associated with poor clinical outcomes, even when disease-related factors are accounted for [11]. Children with both epilepsy and intellectual impairment exhibit higher rates of treatment-resistant seizures [11–13], often leading to anti-seizure medicine (ASM) polypharmacy. They are more prone to both psychiatric [5, 14, 15] and medical comorbidity [5, 16], resulting in medication regimens beyond ASMs. There are higher mortality rates, including sudden death in epilepsy, in people with comorbid intellectual impairment compared to those with only epilepsy [17, 18].

Studies consistently show that intellectual impairment is a common comorbidity among children with epilepsy, though reported prevalence varies widely across settings. Research from high-income countries indicates prevalence rates of 49.1% in Korea [19], 40% in the UK [20], 37.2% in Scotland [21], and 40% in the USA [22]. In African studies, similarly high but more variable rates have been documented, including 18% and 42.5% in Nigeria [23, 24], and 69% in Tanzania [25]. These findings highlight substantial variation, likely reflecting differences in study populations and methods. However, there is limited data on intellectual impairment in children with epilepsy in Uganda. This study aims to address this gap by determining the prevalence of intellectual impairment among children and adolescents with epilepsy in two national referral hospitals in Uganda and examining its association with sociodemographic, perinatal, developmental, and epilepsy-related factors.

## Methods

### Study design and setting

This was a cross-sectional study conducted at two national referral hospitals in Uganda: Mulago National Referral Hospital and Butabika National Referral Mental Hospital. Both hospitals are in Kampala, Uganda’s capital. In Uganda, epilepsy is commonly regarded as a mental health condition and classified by the Ministry of Health under mental, neurological, and substance use disorders [26]. Consequently, many children and adolescents with epilepsy receive care primarily from mental health facilities.

At Mulago Hospital, participants were enrolled from the pediatric neurology and mental health clinics. The pediatric neurology clinic, which operates twice a week and serves children with various neurological disorders from across the country, attends to 50–80 children and adolescents aged 2 months to 17 years each clinic day, with epilepsy accounting for 60% of the cases. The patients are attended to by pediatric residents, pediatricians, and pediatric neurologists. The mental health clinic at Mulago National Referral Hospital serves 20–40 patients daily, with epilepsy accounting for 40–60% of the caseload. This clinic is run by psychiatrists, psychiatry residents, and psychiatric clinical officers (health workers with a diploma in psychiatry).

At Butabika Hospital, participants were enrolled from the Child and Adolescent Mental Health Clinic. This clinic operates from Monday to Friday and serves an average of 250 to 300 children and adolescents each week. Epilepsy cases account for 50–60% of the cases, with approximately 25% newly diagnosed each month.

### Study participants

We enrolled children and adolescents aged 5–17 years with epilepsy who had parental consent, as well as those aged 8 years and above who provided their own assent to participate in the study. In the clinics where participants were enrolled, epilepsy diagnoses documented in the clinic charts are made by psychiatrists, pediatricians, psychiatry residents, or pediatric residents following a comprehensive clinical history and examination, and, where indicated, supportive investigations such as electroencephalography (EEG). Children and adolescents with hearing, vision, or physical impairments that could interfere with their ability to respond to certain study tools were excluded.

### Sample size and sampling technique

The sample size was determined using the Kish-Leslie formula for a single proportion [27]. With a 95% confidence level, an expected prevalence of intellectual impairment of 50% (given the absence of similar studies in our setting), and a precision of 5%, the minimum required sample size was 385 participants. Consecutive sampling was employed to enroll all children and adolescents who met the eligibility criteria during the study period, continuing until the desired sample size was achieved.

### Study variables and measures

#### Dependent variable

The dependent variable in this study was intellectual impairment. Intellectual functioning was assessed using the Raven’s Progressive Matrices (RPM). The RPM is a nonverbal standardized test that measures mental abilities associated with abstract reasoning and problem-solving [28]. The test consists of increasingly difficult pattern-matching tasks. Children aged 5 to 11 years completed the Raven’s Colored Progressive Matrices (CPM), while those aged 12 to 17 years completed the Raven’s Standard Progressive Matrices (SPM). Due to the lack of established norms for the Ugandan setting, we utilized norms for South African children and adolescents [29, 30]. Scores were categorized into five grades based on percentiles: intellectually superior, above-average intellectual capacity, average intellectual capacity, below-average intellectual capacity, and intellectually impaired.

#### Independent variables

The independent variables included sociodemographic variables, perinatal and developmental variables, epilepsy related variables, and behavioral problems.

Sociodemographic factors included the child’s age, sex, education status, primary caregiver, parents’ level of education, parents’ employment status, and family history of mental illness, substance use, or epilepsy.

Perinatal and developmental factors included parental age at the child’s birth, birth weight, preterm birth, maternal fever during pregnancy, place of birth, delay in breastfeeding initiation, crying at birth, neonatal admission, and achievement of developmental milestones (speech and walking). Low birth weight was defined as birth weight below 2.5 kg [31] and delayed breastfeeding as initiation more than 24 h after birth. Delayed speech was defined as the first spoken word after 18 months [32, 33] and delayed walking as taking the first independent steps after 18 months [34].

Epilepsy-related factors included the child’s age at first seizure, duration of treatment, number of seizures in the last three months, and current anti-epileptic medications.

Behavioral problems were assessed using the Strengths and Difficulties Questionnaire- Parent Version (SDQ). It consists of 25 items grouped into five subscales: emotional symptoms, conduct problems, hyperactivity, peer relationship problems, and prosocial behavior. A Total Difficulties Score of 17 or higher was classified as indicating a behavioral problem, 14–16 as borderline, and below 14 as normal. This tool has previously been used to assess for behavioral problems in Uganda in children with epilepsy [35] and in the general population [36].

#### Study procedures

Study participants were enrolled between August 2024 and May 2025. Participants who met the eligibility criteria were approached for participation upon presenting for their clinic visits. The diagnosis of epilepsy was initially obtained from patient records and subsequently confirmed by research assistants through history-taking. The definition of epilepsy, according to the International League Against Epilepsy, as updated in 2014, was used [37]. After obtaining written consent and assent (where applicable), study measures were administered in either English or Luganda (the most common local language in the central region). Participants underwent interviews and completed questionnaires administered by research assistants, who were psychiatric clinical officers, to collect data on sociodemographic characteristics, epilepsy-related factors, perinatal history, and behavioral problems.

### Ethical considerations

This study received ethical approval from the Makerere University School of Medicine Research Ethics Committee (Ref. Mak-SOMREC-2024-889) and the Uganda National Council of Science and Technology (Ref. HS4589ES). Written informed consent was obtained from the caregivers of the study participants, and participants aged 8 to 17 years provided written assent. Children and adolescents identified to have intellectual impairment were referred to a psychiatrist for further assessment and management. This study was conducted in compliance with the ethical guidelines of the 1964 Declaration of Helsinki and its later amendments.

### Data analysis

Data analysis was conducted using STATA version 17.0. Categorical variables were summarized as frequencies and percentages, while continuous variables were described using the mean and standard deviation (SD) or median and interquartile range (IQR) after the Shapiro-Wilks test, and histograms were applied to evaluate the assumption of normality. To assess differences between participants with and without intellectual impairment, Chi-square tests were used for categorical data, Student’s T-tests for normally distributed continuous data, and the Mann-Whitney U test for skewed continuous data. Binary logistic regression was utilized to identify factors associated with intellectual impairment. Variables found to be significant in the bivariate logistic analysis were assessed for multicollinearity using variance inflation factors (VIF). Those with a VIF of less than 3 were included in the final multivariable logistic regression model, controlling for age and gender. We subsequently assessed an interaction term between age group and treatment duration. Statistical significance was determined at a *P*-value of less than 0.05 within a 95% confidence interval.

## Results

### Participant characteristics

#### Sociodemographic characteristics

The study included 386 participants, with 73.6% enrolled at Mulago Hospital. The participants were predominantly male (64.5%), with a mean age of 10 years (SD 3.8). The proportion attending school was 62.7%, and 2.1% were in special needs schools. Regarding family background, 39.3% reported a family history of mental illness and 31.9% a family history of epilepsy. Regarding parental education level, 48.7% of fathers and 48.7% of mothers had attained secondary education, while 21.7% of fathers and 14.6% of mothers had tertiary education. The rest of the demographic characteristics are presented in Table 1.

**Table 1:** Sociodemographic characteristics of study participants

Variable	Frequency	Percentage (%)
**Age **(years, mean [SD])	10 (3.8)	
**Age-group**		
5–11	254	65.8
12–17	132	34.2
**Sex**		
Male	249	64.5
Female	137	35.5
**Study site**		
Butabika Hospital	102	26.4
Mulago Hospital	284	73.6
**Primary caregiver**		
Both parents	177	45.8
Father only	19	4.9
Mother only	123	31.9
Other relative*	66	17.1
Non-relative^§^	1	0.3
**Orphanhood**		
No	376	97.4
Yes	10	2.6
**School attendance**		
No	144	37.3
Yes	242	62.7
**Type of school** (*n* = 242)		
Regular	234	60.6
Special needs	8	2.1
**Family history of mental illness** (*n* = 382)		
No	232	60.7
Yes	150	39.3
**Family history of epilepsy** (*n* = 385) No Yes		
	262	68.1
	123	31.9
**Father’s education level** (*n* = 355)		
No education	16	4.5
Primary	89	25.1
Secondary	173	48.7
Tertiary	77	21.7
**Mother’s education level** (*n* = 378)		
No education	13	3.4
Primary	126	33.3
Secondary	184	48.7
Tertiary	55	14.6
**Father’s employment status** (*n* = 327)		
Unemployed	38	11.6
Employed	289	88.4
**Mother’s employment status** (*n* = 363)		
Unemployed	89	24.5
Employed	274	75.5

*Other relatives included grandmother, aunt and sister

^§^Non-relatives included foster mother

#### Perinatal characteristics, epilepsy related characteristics, and behavioral problems

Perinatal history revealed that 6.6% were born preterm and 10.3% had low birth weight. Nearly a third required neonatal unit admission (31.0%) and a similar proportion experienced delayed breastfeeding. Developmentally, 39.4% of the children had delayed speech, and 28.1% had delayed walking.

Early childhood onset of epilepsy (> 1-<6 years) occurred in 47.6%, and 41.7% had experienced seizures for more than two years. Regarding seizure frequency, 25.3% had more than 10 seizures in the preceding three months. Over 90% were receiving antiseizure medication (94.5%), 78.7% having been on it for five years or less. ASM polypharmacy was present in 34.6% of cases.

Regarding behavioral problems, over a third (36.8%) had SDQ scores in the abnormal range, while 20.4% were borderline. These findings are presented in Table 2.

**Table 2:** Perinatal characteristics, epilepsy-related characteristics and behavioral problems in study participants

Variable	Frequency	Percentage (%)
**Perinatal and Development Variables**		
**Preterm birth** (*n* = 364)		
Yes	24	6.6
**Birth weight** (kg, mean [SD])	3.1 (0.7)	
**Low birth weight** (*n* = 311)		
Yes	32	10.3
**Fever during pregnancy for the mother** (*n* = 333)		
Yes	65	19.5
**Birth in hospital** (*n* = 376)		
Yes	346	92.0
**Child cried at birth** (*n* = 342)		
Yes	248	72.5
**Neonatal unit admission after birth** (*n* = 348)		
Yes	108	31.1
**Delayed breastfeeding** (*n* = 342)		
Yes	112	32.8
**Ability to speak** (*n* = 385)		
Yes	293	75.9
**Age at first words **(months, median [IQR])	11 (9–18)	
**Delayed speech** (*n* = 361)		
Yes	152	39.4
**Ability to walk** (*n* = 385)		
Yes	340	88.1
**Age at first independent steps **(months, median [IQR])	12 (9–18)	
**Delayed walking** (*n* = 357)		
Yes	108	28.1
**Paternal age-group at child’s birth **(years, *n* = 338)		
Late-adolescence/young adulthood (17–34)	208	61.5
Mid adulthood (34–40)	76	22.5
Advanced paternal age (40–65)	54	15.9
**Maternal age-group at child’s birth** (years, *n* = 356)		
Adolescence (14–17)	10	2.8
Young adulthood (18–34)	300	84.3
Advanced maternal age (35–43)	46	12.9
**Epilepsy Characteristics and Treatment**		
**Age of epilepsy onset (years)** (*n* = 372)		
Neonate and infancy	91	24.5
Early childhood (1–5)	177	47.6
Late childhood (6–11)	72	19.4
Adolescence (12–17)	32	8.6
**Seizures ≥ 2 years**		
Yes	155	41.7
**Number of seizures (past 3 months)**		
0–5	227	60.5
6–10	53	14.1
>10	95	25.3
**On anti-seizure medications** (*n* = 385)		
Yes	380	94.5
**Duration of treatment **(years, *n* = 381)		
≤5years	300	78.7
>5years	81	21.3
**Polypharmacy** (*n* = 379)		
Yes	131	34.6
**Behavioural Problems**		
**Total SDQ score** (Mean [SD])	15 (5.3)	
**Level of behavioural problems**		
Normal	165	42.8
Borderline	79	20.4
Abnormal	142	36.8

### Prevalence of intellectual impairment and distribution of intellectual functioning

The overall prevalence of intellectual impairment was 34.7% (*n* = 134). Participants were next most frequently classified as below average (32.4%) and average (26.4%), with smaller proportions falling into the above-average (5.7%) and superior (0.8%) categories. There was no statistical difference in the distribution of intellectual performance categories between males and females (χ² = 4.53, *P* = 0.339).

### Distribution of study variables across the presence of intellectual impairment

Intellectual impairment was more prevalent among participants from Mulago than Butabika Hospital (37.7% vs. 26.5%; *P* = 0.041), those cared for by mothers only (*P* = 0.022), not attending school (70.1% vs. 13.6%) or attending special needs schools (*P* < 0.001), and those whose fathers (43.7% vs. 15.6%; *P* < 0.001) or mothers (53.8% vs. 20.0%; *P* = 0.026) had no formal education.

Perinatal factors that were more prevalent in children with intellectual impairment included low birth weight (2.9 kg vs. 3.2 kg, *P* = 0.003), absence of crying at birth (*P* = 0.004), neonatal admission (*P* = 0.001), and delayed breastfeeding initiation (*P* < 0.001).

Developmentally, children with intellectual impairment were more likely to have experienced speech delays (*P* < 0.001) and motor delays (*P* < 0.001), with later first words (18 months vs. 10 months) and first independent steps (16 months vs. 11 months).

Intellectual impairment was higher in participants with earlier epilepsy onset (*P* < 0.001), seizure onset before two years (45.8% vs. 26.7%, *P* < 0.001), treatment for more than five years (54.3% vs. 29.3%, *P* < 0.001), and ASM polypharmacy (46.6% vs. 27.8%, *P* < 0.001). Behavioral problems were also more common (50.7% vs. 21.2%, *P* < 0.001). (Table 3)

**Table 3:** Differences in participant characteristics across the presence of intellectual impairment

Variable	Intellectual impairment *n* (%)		z/t/ χ^2^	*P*-value
	No	Yes		
	252 (65.3)	134 (34.7)		
**Sociodemographic Variables**				
**Age **(years, mean [SD])	9.7 (3.8)	10.3 (0.3)	-1.46	0.143
**Age-group**				
5–11	172 (68.3)	82 (61.2)	1.94	0.164
12–17	80 (31.7)	52 (38.8)		
**Sex**				
Male	165 (65.4)	84 (62.7)	0.29	0.586
Female	87 (34.5)	50 (37.3)		
**Study site**				
Butabika NRMH	75 (29.7)	27 (20.1)	4.16	**0.041**
Mulago NRH	177 (70.2)	107 (79.9)		
**Primary caregiver**				
Both parents	130 (51.2)	47 (35.1)	11.39	**0.022**
Father only	13 (5.2)	6 (4.5)		
Mother only	71 (28.2)	52 (38.8)		
Other relative	37 (14.7)	29 (21.6)		
Non-relative	1 (0.4)	0 (0.0)		
**Orphanhood**				
No	245 (97.2)	131 (97.8)	0.10	0.751
Yes	7 (2.8)	3 (2.2)		
**School attendance**				
No	43 (17.1)	101 (75.4)	127.17	**< 0.001**
Yes	209 (82.9)	33 (24.6)		
**Type of school**				
Regular	208 (82.5)	26 (19.4)	146.14	**< 0.001**
Special needs	2 (0.8)	6 (4.5)		
Not in school	42 (16.7)	102 (76.1)		
**Family history of mental illness** (*n* = 382)				
No	145 (58.2)	87 (65.4)	1.87	0.171
Yes	104 (41.8)	46 (35.4)		
**Family history of epilepsy** (*n* = 385)				
No	163 (64.9)	99 (73.9)	3.21	0.073
Yes	88 (35.1)	35 (26.1)		
**Father’s education level** (*n* = 355)				
No education	9 (3.9)	7 (5.7)	**21.54**	**< 0.001**
Primary	45 (19.4)	44 (35.8)		
Secondary	113 (48.7)	60 (48.8)		
Tertiary	65 (28.0)	12 (9.8)		
**Mother’s education level** (*n* = 378)				
No education	6 (2.4)	7 (5.3)	**9.29**	**0.026**
Primary	75 (30.4)	51 (38.9)		
Secondary	122 (49.4)	62 (47.3)		
Tertiary	44 (17.8)	11 (8.4)		
**Father’s employment status** (*n* = 327)				
Unemployed	24 (10.8)	14 (13.3)	0.44	0.506
Employed	198 (89.2)	91 (86.7)		
**Mother’s employment status** (*n* = 363)				
Unemployed	52 (21.8)	37 (29.6)	2.66	0.103
Employed	186 (78.1)	88 (70.4)		
**Perinatal Variables**				
**Preterm birth** (*n* = 364)				
No	226 (94.6)	114 (91.2)	1.51	0.220
Yes	13 (5.4)	11 (8.8)		
**Birth weight **(kg, mean [SD])	3.2 (0.1)	2.9 (0.1)	3.03	**0.003**
**Low birth weight** (*n* = 311)				
No	186 (91.2)	93 (86.9)	1.38	0.240
Yes	18 (8.8)	14 (13.1)		
**Fever during pregnancy for the mother** (*n* = 333)				
No	176 (80.7)	92 (80.0)	0.03	0.872
Yes	42 (19.2)	23 (20.0)		
**Birth in hospital** (*n* = 376)				
No	16 (6.5)	14 (10.9)	2.21	0.137
Yes	231 (93.5)	115 (89.1)		
**Child cried at birth** (*n* = 342)				
No	50 (22.4)	44 (37.0)	8.25	**0.004**
Yes	173 (77.6)	75 (63.0)		
**Neonatal unit admission after birth** (*n* = 348)				
No	170 (74.9)	70 (57.9)	10.71	**0.001**
Yes	57 (25.1)	51 (42.1)		
**Delayed breastfeeding initiation** (*n* = 342)				
No	165 (74.7)	65 (53.7)	15.56	**< 0.001**
Yes	56 (25.3)	56 (46.3)		
**Ability to speak**				
No	17 (6.7)	76 (56.7)	119.44	**< 0.001**
Yes	235 (93.3)	58 (43.3)		
**Age at first words **(months, median [IQR])	10 (9–18)	18 (9–24)	**-**2.98	**0.003**
**Delayed speech** (*n* = 361)				
No	177 (76.6)	32 (24.6)	92.30	**< 0.001**
Yes	54 (23.4)	98 (75.4)		
**Current ability to walk**				
No	1 (0.4)	45 (33.6)	91.77	**< 0.001**
Yes	251 (99.6)	89 (66.4)		
**Age at first independent steps** (months, median [IQR])	11 (9–18)	16 (10–24)	-4.27	**< 0.001**
**Delayed walking** (*n* = 357)				
No	190 (83.0)	59 (46.1)	52.87	**< 0.001**
Yes	39 (17.0)	69 (53.9)		
**Paternal age at child’s birth **(years, mean [SD])	33 (0.5)	33 (0.7)	-0.16	0.872
**Paternal age-group at child’s birth (years)** (*n* = 338)				
late-adolescence/young adulthood (17–34)	135 (60.8)	73 (62.9)	3.86	0.146
Mid adulthood (34–40)	56 (25.2)	20 (17.3)		
Advanced paternal age (40–65)	31 (13.9)	23 (19.8)		
**Maternal age at child’s birth** (years, mean [SD])	27 (0.4)	27 (0.5)	-0.55	0.584
**Maternal age-group at child’s birth (years)** (*n* = 356)				
Adolescence (14–17)	7 (3.0)	3 (2.4)	0.97	0.616
Young adulthood (18–34)	197 (85.3)	103 (82.4)		
Advanced maternal age (35–43)	27 (11.7)	19 (15.2)		
**Epilepsy Characteristics and Treatment**				
**Age of epilepsy onset (years)** (*n* = 372)				
Neonate and infancy	46 (18.9)	45 (34.9)	23.06	**< 0.001**
Early childhood (1–5)	112 (46.1)	65 (50.4)		
Late childhood (6–11)	56 (23.0)	16 (12.4)		
Adolescence (12–17)	29 (12.0)	3 (2.3)		
**Seizures below 2 years**				
No	159 (65.4)	58 (45.0)	14.52	**< 0.001**
Yes	84 (34.6)	71 (55.0)		
**Number of seizures (past 3 months)**				
0–5	146 (60.6)	81 (60.4)	0.12	0.940
6–10	35 (14.5)	18 (13.4)		
>10	60 (24.9)	35 (26.2)		
**Current anti-seizure medication use**				
No	39 (15.5)	3 (2.2)	0.62	0.428
Yes	213 (84.5)	131 (97.8)		
**Duration of treatment (years)** (*n* = 381)				
≤5years	212 (85.1)	88 (66.7)	17.58	**< 0.001**
>5years	37 (14.9)	44 (33.3)		
**Anti-seizure medication polypharmacy** (*n* = 379)				
No	179 (71.9)	69 (57.5)	13.36	**< 0.001**
Yes	70 (28.1)	51 (42.5)		
**Behavioral Problems**				
**Total SDQ score** (mean [SD])	14 (0.3)	18 (0.5)	-7.53	**< 0.001**
**Level of behavioral problems**				
Normal	130 (51.6)	35 (26.1)	**29.30**	**< 0.001**
Borderline	52 (20.6)	27 (20.1)		
Abnormal	70 (27.8)	72 (53.7)		

### Factors associated with intellectual impairment in children and adolescents with epilepsy: multivariable analysis

Variables that were significant (*P* < 0.05) in the bivariate logistic regressions were included in the model, controlling for age and sex. The bivariate logistic regression findings are in Additional file 1. Variables with a VIF greater than 3 (school attendance, neonatal admission, delayed breastfeeding, ability to speak, SDQ score, and ability to walk) were excluded from the final model, and the final model’s mean VIF was 1.51. The variables included in the final model were age group, sex, primary caregiver, type of school, father’s education level, mother’s education level, birth weight, crying at birth, delayed speech, delayed walking, age of epilepsy onset, seizure onset below 2 years, duration of epilepsy treatment, ASM polypharmacy, and level of behavioral problems. Collinearity diagnostics remained acceptable, and the interaction term was not statistically significant (*P* = 0.921). The final model had a sensitivity of 81.52%, a specificity of 91.11%, a negative predictive value of 90.61%, and correctly classified 82.42% of those with intellectual impairment. The model had good goodness-of-fit for all included variables, with a *P*-value of 0.999.

The likelihood of intellectual impairment increased with age, with children aged 12–17 years being significantly more likely to have intellectual impairment compared to those aged 5–11 years (adjusted odds ratio [aOR] = 6.94, 95% confidence interval [CI] = 2.27–21.27, *P* = 0.001). Additionally, being in a special needs school (aOR = 14.50, CI = 1.65–127.75, *P* = 0.016) and not attending school (aOR = 26.01, CI = 9.35–72.34, *P* < 0.001) were associated with increased odds of intellectual impairment. Among developmental factors, delayed speech was associated with a significantly greater likelihood of intellectual impairment (aOR = 3.26, CI = 1.09–9.65, *P* = 0.033). However, having epilepsy onset during adolescence was protective against intellectual impairment when compared to neonatal or infancy onset (aOR = 0.05, CI = 0.00–0.75, *P* = 0.030). Children on ASM polypharmacy also had an increased likelihood of intellectual impairment (aOR = 3.08, CI = 1.20–7.92, *P* = 0.020). This is presented in Table 4.

**Table 4:** Multivariate logistic analysis of factors associated with intellectual impairment

Variable	Adjusted odds ratio (95% Confidence Interval)	*P*-value
**Age-group**		
5–11	1 (reference)	
12–17	6.94 (2.27–21.27)	**0.001**
**Sex**		
Female	1 (reference)	
Male	0.77 (0.31–1.90)	0.568
**Primary caregiver**		
Both parents	1 (reference)	
Father only	0.34 (0.03–5.02)	0.431
Mother only	1.50 (0.61–3.67)	0.378
Other relative	4.57 (0.91–22.98)	0.065
**Type of school**		
Regular	1 (reference)	
Special needs	14.50 (1.65–127.75)	**0.016**
Not in school	26.01 (9.35–72.34)	**< 0.001**
**Father’s education**		
No education	1 (reference)	
Primary	1.44 (0.24–8.74)	0.691
Secondary	0.81 (0.34–4.80)	0.821
Tertiary	0.98 (0.13–7.51)	0.983
**Mother’s education**		
No education	1 (reference)	
Primary	0.20 (0.01–3.37)	0.273
Secondary	0.29 (0.02–5.33)	0.408
Tertiary	0.29 (0.01–6.97)	0.442
**Birth weight (kg)**	0.59 (0.31–1.12)	0.105
**Child cried at birth**		
No	1 (reference)	
Yes	2.45 (0.81–7.47)	0.115
**Delayed speech**		
No	1 (reference)	
Yes	3.26 (1.09–9.65)	**0.033**
**Delayed walking**		
No	1 (reference)	
Yes	2.14 (0.66–6.91)	0.204
**Age of epilepsy onset (years)**		
Neonate and infancy	1 (reference)	
Early childhood (1–6)	0.37 (0.10–1.42)	0.147
Late childhood (6–11)	0.69 (0.11–4.19)	0.688
Adolescence (12–17)	0.05 (0.00–0.75)	**0.030**
**Seizures below 2 years**		
No	1 (reference)	
Yes	0.87 (0.28–2.81)	0.924
**Duration of treatment (years)**		
≤5 years	1 (reference)	
>5 years	0.95 (0.32–2.81)	0.924
**ASM Polypharmacy**		
No	1 (reference)	
Yes	3.08 (1.20–7.92)	**0.020**
**Level of behavioral problems**		
Normal	1 (reference)	
Borderline	0.92 (0.30–2.80)	0.879
Abnormal	1.76 (0.64–4.84)	0.276

## Discussion

This study assessed the prevalence and associated factors of intellectual impairment in children and adolescents with epilepsy in two national referral hospitals in Uganda. We found a prevalence of 34.7% of intellectual impairment. Older age, not attending school, being in a special needs school, delayed speech, and polypharmacy were positively associated with intellectual impairment, while adolescent epilepsy onset was negatively associated.

The prevalence of impaired intellectual function in our study was 34.7%, indicating that it is a significant comorbidity of pediatric epilepsy in our setting and highlighting the need for targeted interventions and support for children and their families. This relatively high prevalence likely reflects the clinical profile of a tertiary-care cohort, where children with more complex epilepsy and neurodevelopmental comorbidities are preferentially referred. This rate of intellectual impairment is similar to the 37.3% that was reported by Sabaz et al. [21], but lower than the prevalence of 40% in the UK [20], 42.5% in Nigeria [24], 54.7% in Korea [19], and 69% in Tanzania [25]. However, it is higher than the prevalence reported in Nigeria at 18.1% [23] and in the U.S. at 15.4% [22].

Variations across studies may be due to differences in the methods used to assess intellectual functioning, participant age ranges, study settings, and geographic contexts. While we used the Raven’s Progressive Matrices to assess intellectual functioning, most studies employed the Wechsler Intelligence Scales [18, 21–23], and one study utilized the Goodenough–Harris Drawing Test [25]. Differences in sampling frames may have also contributed, as our participants were recruited from clinical settings, while some studies drew participants from community settings [20, 25]. The study in Tanzania that reported a prevalence of intellectual impairment of 69% [25] reported that about 90% of the children were receiving phenobarbital, a medication associated with adverse cognitive function [38]. In contrast, children in the Ugandan national referral hospitals are more commonly treated with sodium valproate and carbamazepine, which are less likely to affect intellectual function negatively [39, 40]. The study in Nigeria that reported an intellectual impairment prevalence of 18.1% [23] included only participants with idiopathic epilepsy and no comorbid neurological diagnosis. In contrast, our study had a mixed population with both idiopathic epilepsy and symptomatic epilepsy, as well as additional neurologic disorders. Because the underlying causes of symptomatic epilepsy and other neurological conditions can independently impair intellectual functioning, this difference in case mix likely accounts for the higher prevalence observed in our study [4, 6].

We found that older age was significantly associated with intellectual impairment. This contrasts with previous studies, which did not report an association between age and intellectual impairment in children with epilepsy [22, 23]. This discrepancy may be due to differences in how age groups were categorized across the studies. Although older age may suggest a longer cumulative burden of epilepsy, this explanation remains hypothetical because our interaction analysis showed that duration of treatment did not modify the association between age group and intellectual impairment. This implies that the age effect may instead reflect unmeasured factors such as delayed diagnosis, earlier periods of poorly controlled seizures, or unmet developmental needs, rather than a confirmed cumulative effect. Given our cross-sectional design, these findings represent associations rather than causal relationships. Cohort studies of children with epilepsy after disease onset have suggested the existence of a slow progressive impact on cognition over time, and this may explain the association of older age and intellectual impairment [22, 41].

Regarding school placement, children and adolescents with intellectual impairment were likely to not be attending school. School-related challenges are well-documented among children with epilepsy, with studies showing that these children often begin school later, miss more school days, repeat grades more frequently, and, in many cases, do not attend school at all [42–44]. Several factors may contribute to these difficulties, including poorly controlled seizures, stigma, parental attitudes, and comorbid intellectual impairment [42, 43, 45]. The high likelihood of non-attendance among children with intellectual impairment may further reflect stigma, limited availability of special needs schools, high school fees, and logistical barriers such as transportation to and from school [46]. Attending a special needs school was also associated with intellectual impairment, consistent with findings from other studies [41]. Due to persistent learning difficulties, children with epilepsy and intellectual impairment often need additional learning support and special educational services [47].

Children and adolescents with intellectual impairment were more likely to have delayed speech than their peers without intellectual impairment. Both epilepsy and intellectual disability independently hinder speech and language development [48]. Children with epilepsy are at risk of speech delay due to shared underlying pathophysiological mechanisms of the disorders or due to abnormal electrical activity during seizures, disrupting neural pathways involved in speech and language development [49]. The cognitive deficits inherent to intellectual impairment can also hinder language acquisition [50]. Consequently, the combined presence of epilepsy and intellectual impairment is likely to exert a greater adverse effect on speech development than epilepsy alone.

Age of seizure onset has been widely associated with intellectual impairment in epilepsy, with several studies identifying earlier onset as a risk factor for intellectual impairment [20, 23, 51]. In this study, adolescent onset of epilepsy was protective against intellectual impairment, reinforcing the notion of a neurodevelopmental impact of epilepsy on cognition. Seizures occurring in early childhood have a higher likelihood of detrimental effects on cognition, as they may disrupt critical processes involved in cognitive development [51]. The proliferation of synapses begins during gestation and peaks within the first two years after birth [52]. Seizures occurring during early childhood, a sensitive period of synaptogenesis and network formation, are more likely to interfere with ongoing neurocognitive development [51]. This is further supported by evidence showing that individuals with early-onset epilepsy tend to exhibit structural brain differences, such as reduced total white matter volume, which has been linked to poorer cognitive outcomes and intellectual impairment [53]. However, many of these critical neurodevelopmental processes are largely completed by adolescence, so the disruptive effect on brain development may be less pronounced than in early childhood.

Polypharmacy for the management of epilepsy was also found to be significantly associated with intellectual impairment. This is consistent with other studies that show that children who require multiple ASMs for seizure control are more likely to have intellectual impairment compared to those on monotherapy [20, 54]. The need for multiple ASMs may be due to treatment-resistant seizures, which have been linked to intellectual impairment in children with epilepsy [11, 13]. It should also be noted that the use of multiple ASMs increases the risk of adverse events, including worsening intellectual impairment [8]. Therefore, clinicians and caregivers should routinely monitor for these effects [55]. However, this association should be interpreted with caution, as the cross-sectional design of this study does not allow determination of the direction of the relationship. Longitudinal studies are needed to clarify causal pathways between polypharmacy and intellectual impairment.

### Strengths and Limitations

To our knowledge, this is the first study in Uganda to assess intellectual functioning in children and adolescents with epilepsy, contributing valuable data on neuropsychiatric comorbidities in this population across the African continent.

However, several limitations should be noted. The cross-sectional design of the study means that data were collected at a single point in time, and this prevents any conclusions about causality. Information on perinatal and epilepsy-related factors was obtained from caregiver reports rather than medical records, potentially introducing recall bias.

Intellectual functioning was assessed using the Raven’s Progressive Matrices, which has not been validated in Uganda, and no locally standardized cut-offs exist. We therefore applied South African cut-offs, which may not accurately represent Ugandan children and adolescents. Although RPM is non-verbal and widely used in multicultural contexts, its interpretation is still influenced by educational exposure, familiarity with abstract pattern-based tasks, and test-taking experience. The use of South African norms may therefore have led to either over- or under-estimation of intellectual impairment in our sample, and some participants, particularly those near the percentile thresholds, may have been misclassified. However, given the non-verbal nature of the RPM, it is also suitable for children with neurodevelopmental comorbidities such as autism spectrum disorder (ASD), which is frequently observed alongside epilepsy and intellectual impairment [56]. Although ASD was not formally assessed in this study, the RPM remains an appropriate tool to capture reasoning ability in this population. Nevertheless, establishing Ugandan-specific RPM norms would improve classification accuracy in future studies.

Children and adolescents with significant sensory or physical impairments that could interfere with their ability to complete the assessment tools were excluded from the study. This exclusion may have introduced selection bias by systematically omitting individuals who are more likely to have more severe forms of intellectual impairment. As a result, the prevalence and severity of intellectual impairment observed in this study may be underestimated.

Finally, the study was conducted at national referral hospitals, which are most likely to serve patients with more severe forms of epilepsy who have been referred from other health facilities. As a result, the high prevalence of intellectual impairment, polypharmacy, and behavioral problems observed in this study likely reflects the severity of epilepsy within a tertiary-care population. These findings should therefore be interpreted as representative of children and adolescents receiving specialist epilepsy care in Uganda and may not be generalizable to those managed in primary health care or community settings. Future studies conducted in community and lower-level health facilities are needed to provide population-level estimates and inform broader public health planning.

## Conclusions

The study found that about a third of the children and adolescents with epilepsy had intellectual impairment, and this was associated with being of older age, delayed speech, ASM polypharmacy, not attending school, and being in a special needs school. Those with adolescent-onset epilepsy were less likely to have cognitive impairment. These findings highlight the importance of intellectual impairment as a significant comorbidity in epilepsy and the need to assess the intellectual function of children with epilepsy to enable timely intervention. Identifying associated factors is clinically important, as they can help guide which patients should be prioritized for assessment and referral to specialized care.

## Supplementary Information

Below is the link to the electronic supplementary material.

Supplementary Material 1

## Data Availability

The datasets analyzed in the current study are not publicly available, as further analyses are being conducted on them, but are available from the corresponding author upon reasonable request.

## Abbreviations

ASM: Anti-seizure medication
aOR: Adjusted odds ratio
CPM: Colored Progressive Matrices
RPM: Raven Progressive Matrices
SPM: Standard Progressive Matrices
SDQ: Strengths and Difficulties Questionnaire
