# Supplementary material for: Prevalence and factors associated with intellectual impairment in children with epilepsy in two national referral hospitals in Uganda
Source: Acta Epileptol. 2026 Jul 1;8:24. doi: 10.1186/s42494-026-00265-3 (PMC13321425; doi:10.1186/s42494-026-00265-3)
Supplement: Supplementary file 1 — Supplementary Material 1 [file 42494_2026_265_MOESM1_ESM.docx]

**INTELLECTUAL IMPAIRMENT AND ASSOCIATED FACTORS IN CHILDREN WITH EPILEPSY IN TWO NATIONAL REFERRAL HOSPITALS IN UGANDA**

**Supplementary Table 1**

**Bivariate logistic regression of factors associated with intellectual impairment in children and adolescents with epilepsy attending two national referral hospitals in Uganda**

| **Variable** | **Intellectual disability** | | **Logistic bivariate analysis** | |
| --- | --- | --- | --- | --- |
|  | **No** | **Yes** |  |  |
|  | **252 (65.3)** | **134 (34.7)** | **Crude odds ratio (95% CI)** | **p-value** |
| **Sociodemographic Variables** | | | | |
| **Age (years)** [mean (SD)] | 9.7 (3.8) | 10.3 (0.3) | 1.04 (0.98-1.10) | 0.143 |
| **Age-group** | | | | |
| 5–11 | 172 (67.7) | 82 (32.3) | 1 (reference) |  |
| 12–17 | 80 (60.6) | 52 (39.4) | 1.36 (0.88-2.11) | 0.165 |
| **Sex** | | | | |
| Male | 165 (66.3) | 84 (33.7) | 1 (reference) |  |
| Female | 87 (63.5) | 50 (36.5) | 0.88 (0.57-1.37) | 0.586 |
| **Study site** | | | | |
| Butabika NRMH | 75 (73.5) | 27 (26.5) | 1 (reference) |  |
| Mulago NRH | 177 (62.3) | 107 (37.7) | 1.68 (1.02-2.77) | **0.043** |
| **Primary caregiver** | | | | |
| Both parents | 130 (73.5) | 47 (26.6) | 1 (reference) |  |
| Father only | 13 (68.4) | 6 (31.6) | 1.28 (0.45 – 3.55) | 0.640 |
| Mother only | 71 (57.7) | 52 (42.3) | 2.03 (1.24 – 3.31) | **0.005** |
| Other relative | 37 (56.1) | 29 (43.9) | 2.17 (1.20 – 3.91) | **0.010** |
| Non-relative | 1 (100.0) | 0 (0.0) | 1 (empty) |  |
| **School attendance** | | | | |
| No | 43 (29.8) | 101 (70.1) | 1 (reference) |  |
| Yes | 209 (86.4) | 33 (13.6) | 0.07 (0.04-0.11) | **<0.001** |
| **Type of school** | | | | |
| Regular | 208 (88.9) | 26 (11.1) | 1 (reference) |  |
| Special needs | 2 (25.0) | 6 (75.0) | 24 (4.60-125.14) | **<0.001** |
| Not in school | 42 (29.2) | 102 (70.8) | 19.42 (11.28-33.45) | **<0.001** |
| **Family history of mental illness** (n=382) | | | | |
| No | 145 (62.5) | 87 (37.5) | 1 (reference) |  |
| Yes | 104 (69.3) | 46 (30.7) | 0.74 (0.48-1.14) | 0.172 |
| **Family history of epilepsy** (n=385) | | | | |
| No | 163 (62.2) | 99 (37.8) | 1 (reference) |  |
| Yes | 88 (71.5) | 35 (28.5) | 0.65 (0.41-1.04) | 0.074 |
| **Father's education** (n=355) | | | | |
| No education | 9 (56.3) | 7 (43.7) | 1 (reference) |  |
| Primary | 45 (50.6) | 44 (49.4) | 1.26 (0.43-3.67) | 0.676 |
| Secondary | 113 (65.3) | 60 (34.7) | 0.68 (0.24-1.92) | 0.470 |
| Tertiary | 65 (84.4) | 12 (15.6) | 0.24 (0.07-0.76) | **0.015** |
| **Mother's education** (n=378) | | | | |
| No education | 6 (46.2) | 7 (53.8) | 1 (reference) |  |
| Primary | 75 (59.5) | 51 (40.5) | 0.58 (0.19-1.83) | 0.356 |
| Secondary | 122 (66.3) | 62 (33.7) | 0.44 (0.14-1.35) | 0.150 |
| Tertiary | 44 (80.0)) | 11 (20.0) | 0.21 (0.06-0.77) | **0.018** |
| **Father’s employment status** (n=327) | | | | |
| Unemployed | 24 (63.2) | 14 (36.8) | 1 (reference) |  |
| Employed | 198 (68.5) | 91 (31.5) | 0.79 (0.38-1.59) | 0.507 |
| **Mother’s employment status** (n=363) | | | | |
| Unemployed | 52 (58.4) | 37 (41.6) | 1 (reference) |  |
| Employed | 186 (67.9) | 88 (32.1) | 0.66 (0.41-1.08) | 0.104 |
| **Perinatal Variables** | | | | |
| **Preterm birth** (n=364) | | | | |
| No | 226 (66.5) | 114 (33.5) | 1 (reference) |  |
| Yes | 13 (54.2) | 11 (45.8) | 1.68 (0.73-3.86) | 0.224 |
| **Birth weight (kg**) [mean (SD)] | 3.2 (0.1) | 2.9 (0.1) | 0.55 (0.37-0.82) | **0.003** |
| **Low birth weight** (n=311) | | | | |
| No | 186 (66.7) | 93 (33.3) | 1 (reference) |  |
| Yes | 18 (56.3) | 14 (43.7) | 1.56 (0.74-3.26) | 0.243 |
| **Fever during pregnancy for mother** (n=333) | | | | |
| No | 176 (65.7) | 92 (34.3) | 1 (reference) |  |
| Yes | 42 (64.6) | 23 (35.4) | 1.05 (0.59-1.85) | 0.872 |
| **Birth in hospital** (n=376) | | | | |
| No | 16 (53.3) | 14 (46.7) | 1 (reference) |  |
| Yes | 231 (66.7) | 115 (33.2) | 0.57 (0.27-1.21) | 0.141 |
| **Child cried at birth** (n=342) | | | | |
| No | 50 (53.2) | 44 (46.8) | 1 (reference) |  |
| Yes | 173 (69.7) | 75 (30.2) | 0.49 (0.30-0.80) | **0.004** |
| **Neonatal unit admission after birth** (n=348) | | | | |
| No | 170 (70.8) | 70 (29.2) | 1 (reference) |  |
| Yes | 57 (52.8) | 51 (47.2) | 2.17 (1.36-3.47) | **<0.001** |
| **Delayed breastfeeding** (n=342) | | | | |
| No | 165 (71.7) | 65 (28.3) | 1 (reference) |  |
| Yes | 56 (50.0) | 56 (50.0) | 2.54 (1.59-4.06) | **<0.001** |
| **Ability to speak** | | | | |
| No | 17 (18.3) | 76 (81.7) | 1 (reference) |  |
| Yes | 235 (80.2) | 58 (19.8) | 0.06 (0.03-0.100) | **<0.001** |
| **Age at first words (months)** [median (IQR)] | 10 (9 - 18) | 18 (9 - 24) | 1.04 (1.01-1.07) | **<0.001** |
| **Delayed speech** (n=361) | | | | |
| No | 177 (84.7) | 32 (15.3) | 1 (reference) |  |
| Yes | 54 (35.5) | 98 (64.5) | 10.04 (6.07-16.58) | **<0.001** |
| **Ability to walk** | | | | |
| No | 1 (2.2) | 45 (97.8) | 1 (reference) |  |
| Yes | 251 (73.8) | 89 (26.2) | 0.01 (0.00-0.06) | **<0.001** |
| **Age at first independent steps (months)** [median (IQR)] | 11 (9 - 18) | 16 (10 - 24) | 1.06 (1.03-1.09) | **<0.001** |
| **Delayed walking** (n=357) | | | | |
| No | 190 (76.3) | 59 (23.7) | 1 (reference) |  |
| Yes | 39 (64.2) | 69 (63.9) | 5.69 (3.49-9.29) | **<0.001** |
| **Paternal age at child’s birth (years)** [mean (SD)] | 33 (0.5) | 33 (0.7) | 1.00 (0.97-1.03) | 0.872 |
| **Paternal age-group at child’s birth (years)** (n=338) | | | | |
| late-adolescence/young adulthood (17-34) | 135 (60.8) | 73 (62.9) | 1 (reference) |  |
| Mid adulthood (34-40) | 56 (25.2) | 20 (17.2) | 0.66 (0.37-1.18) | 0.164 |
| Advanced age (40-65) | 31 (13.9) | 23 (19.8) | 1.37 (0.75-2.52) | 0.309 |
| **Maternal age at child’s birth (years)** [mean (SD)] | 27 (0.4) | 27 (0.5) | 1.01 (0.98-1.05) | 0.583 |
| **Maternal age-group at child’s birth (years)** (n=356) | | | | |
| Adolescence (14-17) | 7 (3.0) | 3 (2.4) | 1 (reference) |  |
| Young adulthood (18-34) | 197 (85.3) | 103 (82.4) | 1.22 (0.31-4.82) | 0.777 |
| Advanced age (35-43) | 27 (11.7) | 19 (15.2) | 1.64 (0.38-7.17) | 0.510 |
| **Epilepsy Characteristics and Treatment** | | | | |
| **Age of epilepsy onset (years)** (n=372) | | | | |
| Neonate and infancy | 46 (50.6) | 45 (49.5) | 1 (reference) |  |
| Early childhood (>1-<6) | 112 (63.3) | 65 (36.7) | 0.59 (0.36-0.99) | **0.046** |
| Late childhood (6-11) | 56 (77.8) | 16 (22.2) | 0.29 (0.14-0.58) | **<0.001** |
| Adolescence (12 - 17) | 29 (90.6) | 3 (9.4) | 0.11 (0.03-0.37) | **<0.001** |
| **Seizures before 2 years** | | | | |
| No | 159 (73.3) | 58 (26.7) | 1 (reference) |  |
| Yes | 84 (54.2) | 71 (45.8) | 2.32 (1.49-3.58) | **<0.001** |
| **Number of seizures in past 3 months** | | | | |
| 0–5 | 146 (64.3) | 81 (35.7) | 1 (reference) |  |
| 6–10 | 35 (66.0) | 18 (33.9) | 0.93 (0.49-1.74) | 0.814 |
| >10 | 60 (63.2) | 35 (35.8) | 1.05 (0.64-1.73) | 0.843 |
| **On anti-seizure medication** | | | | |
| No | 39 (50.0) | 3 (50.0) | 1 (reference) |  |
| Yes | 249 (65.5) | 131(34.7) | 0.53 (0.10-2.64) | 0.435 |
| **Duration of treatment (years)** (n=381) | | | | |
| </=5years | 212 (70.7) | 88 (29.3) | 1 (reference) |  |
| >5years | 37 (45.7) | 44 (54.3) | 2.86 (1.73-4.73) | **<0.001** |
| **Polypharmacy** (n=379) | | | | |
| No | 179 (72.2) | 69 (27.8) | 1 (reference) |  |
| Yes | 70 (53.4) | 51 (46.6) | 2.26 (1.45-3.52) | **<0.001** |
| **Behavioural Problems** | | | | |
| **Total SDQ score** | 14 (0.3) | 18 (0.5) | 1.16 (1.11-1.22) | **<0.001** |
| **Level of behavioural problems** | | | | |
| Normal | 130 (78.8) | 35 (21.2) | 1 (reference) |  |
| Borderline | 52 (65.8) | 27 (34.2) | 1.93 (1.06-3.50) | **0.031** |
| Abnormal | 70 (49.3) | 72 (50.7) | 3.82 (2.32-6.28) | **<0.001** |
